# Impact of cardiovascular comorbidity on increased neutrophil-lymphocyte ratio in pseudoexfoliation syndrome

**DOI:** 10.1097/MD.0000000000029602

**Published:** 2022-07-15

**Authors:** Hea Young Oh, Mee Yon Lee, Young Chun Lee, Hye-Young Shin

**Affiliations:** a Department of Ophthalmology, Uijeongbu St. Mary’s Hospital, College of Medicine, The Catholic University of Korea, Seoul, Republic of Korea.

**Keywords:** cardiovascular comorbidity, cardiovascular disease, neutrophil-lymphocyte ratio, pseudoexfolation syndrome

## Abstract

**Background:**

The purpose of this study was to compare neutrophil-to-lymphocyte ratios (NLRs) of patients with pseudoexfoliation syndrome (PEX) according to the presence of cardiovascular disease (CVD) with those without CVD as controls.

**Methods:**

A total of 197 participants (97 patients with PEX and 100 participants without PEX regarded as the control group) were included in this retrospective study. The PEX group was divided into 2 subgroups, PEX with CVD (group 1) and PEX without CVD (group 2). NLRs were then compared to those of the control group.

**Results:**

The incidence of CVD was significantly (*P* = .015) higher in the PEX group than in the control group. NLR was significantly higher in the group 1 and group 2 compared with that of the control group (*P* = .048 and *P* = .002, respectively). In the PEX group, group 1 showed higher NLR than group 2 (*P* = .023).

**Conclusion:**

Although the PEX group showed a higher incidence of CVD, the NLR was higher in the PEX group regardless of cardiovascular comorbidity than that in the control group.

## 1. Introduction

Pseudoexfoliation syndrome (PEX) occurs mainly in elderly patients with the deposition of abnormal extracellular fibrillar materials in tissues of anterior segments of the eye, such as the lens, iris, ciliary zonules, trabecular meshwork, and corneal endothelium.^[1,2]^ The pathophysiology of PEX is not fully understood but it is assumed that there is a genetic link to the gene lysyl oxidase-like 1, which plays a role in the cross-linking of collagen and elastin in the extracellular matrix. In addition to genetic factors, factors such as oxidative stress and local inflammation in tissues are also associated with the pathogenesis of PEX. Chronic inflammation could trigger proinflammatory cytokines and growth factors such as interleukin-6, interleukin-8, transforming growth factor beta, vascular endothelial growth factor and platelet-derived growth factor, accelerating fibrous tissue production in visceral organs and vessel walls, and leading to systemic diseases as well as ocular manifestations.^[3–6]^

Studies have shown that patients with PEX have an increased risk of systemic vascular diseases,^[7–12]^ including cardiovascular diseases (CVD), coronary heart diseases, cerebrovascular diseases, aortic aneurysm, and peripheral vascular diseases, although the mechanism of association between PEX and vascular diseases remains unclear. A few studies have suggested that pseudoexfoliative materials can cause basement membrane and endothelial dysfunction, leading to fibrillopathy and oxidative stress of vascular walls.^[7,9,11]^

The neutrophil-to-lymphocyte ratio (NLR) has been studied as a biomarker of subclinical inflammation. The role of NLR in CVD, cancers, and autoimmune and inflammatory diseases has been emphasized due to its advantage of being easily identified from complete blood count of peripheral blood.^[10,13]^ NLR has also been reported to play a role in several ocular diseases such as dry eye disease, keratoconus, age-related macular degeneration, diabetic retinopathy, retinal vein occlusion, ischemic optic neuropathy, primary open-angle glaucoma,^[14–16]^ and PEX.^[17–19]^ To the best of our knowledge, there have been 2 reports on the association of elevated NLR in PEX.^[17,18]^ They noted that NLR may act as an effective biomarker indicating subclinical inflammation in PEX. However, previous studies did not exclude other risk factors that could increase the NLR such as CVD.

Thus, it is necessary to check whether NLR level is high even after excluding these risk factors. In this study, we aimed to assess whether NLR is higher in PEX patients regardless of cardiovascular morbidity than in controls.

## 2. Materials and Methods

This study was conducted according to the Declaration of Helsinki. It was approved by the Institutional Review Board of the Uijeongbu St.Mary’s Hospital of Korea. Medical records of a total of 197 subjects who visited the hospital from January 2014 to June 2021 were reviewed retrospectively. The diagnosis of PEX was based on slit-lamp examination conducted by one glaucoma specialist to determine the presence of the deposition of typical PEX materials on the pupillary margin or on the anterior lens capsule after mydriasis. The control group consisted of subjects who underwent an ophthalmological examination before a cataract surgery at this hospital without evidence of PEX material deposition in the anterior segment of the eye. There were 97 patients with PEX and 100 subjects in the control group. PEX patients included 79 with CVD (group 1) and 18 without CVD (group 2). All 197 subjects underwent a complete ophthalmological examination including visual acuity, intraocular pressure measurement, slit-lamp, and fundus examination. Diabetes, hypertension, CVD, systemic, and ocular history were investigated through medical records. Autoimmune and inflammatory diseases, acute/chronic infection, hematologic disorders, and malignancies were excluded from this study. Patients with neutrophilia, neutropenia, lymphocytosis, lymphopenia, ocular surgery, or history of ocular drug use except cataracts were also excluded. Venous blood samples were obtained from all subjects. Complete blood count was performed. Levels of white blood cells, neutrophils, and lymphocytes were measured. NLR was calculated as the ratio of neutrophil count to lymphocyte count.

All statistical analyses were conducted with SPSS 22.0 software (IBM Corp., Armonk, NY). Comparison between groups was made using Student’s *t* test or Mann–Whitney *U* test for continuous variables and χ^2^ test for categorical variables. The Kolmogorov-Smirnov test was performed for those with normal distribution. Levene’s Homogeneity of Variation test was performed to confirm homogeneity. One-way analysis of variance (ANOVA) for parametric values or Kruskal-Wallis test for nonparametric values was used for comparison of >3 groups. If there was a significant difference, pairwise comparisons were done by Student’s *t*-test or Mann–Whitney *U* test with Bonferroni correction to establish the difference between subgroups. Two-way ANOVA was performed to compare the value of NLR between groups on 2 independent factors, PEX and CVD. Tukey post hoc test was used to determine the significance of pair-wise comparisons. Data are described as mean ± standard deviation. In all cases, *P* < .05 was considered statistically significant.

## 3. Results

A total of 197 subjects were screened. There were 97 patients with PEX and 100 subjects without PEX in the control group. There was no significant difference in clinical characteristics such as age, gender, diabetes, or hypertension between PEX patients and control group. However, the incidence of CVD was higher in PEX patients than in the control group (*P* = .015; Table 1). When PEX patients were divided into groups 1 and 2 according to the presence of CVD, there was no difference in clinical characteristics such as age, gender, diabetes, or hypertension between the 2 groups (Table 1). There was no significant difference in WBC or neutrophil count between PEX patients and controls. However, lymphocyte count was higher in PEX patients than in controls (Table 2).

**Table 1:** Clinical characteristics of subjects with pseudoexfoliation syndrome and those in the control group.

Characteristics	Control (n = 100)	PEX (n = 97)	*P* value	PEX without CVD; group 1 (n = 79)	PEX with CVD; group 2 (n = 18)	*P* value
						Control vs group 1 vs group 2
Age (years)	74.73 ± 6.60	76.58 ± 7.69	.072	76.03 ± 7.96	79.00 ± 5.95	.139
Gender (male) (n, %)	47 (47.0)	48 (49.5)	.727	41 (51.9)	7 (38.9)	.572
Diabetes Mellitus (n, %)	29 (29.0)	36 (37.1)	.226	26 (32.9)	10 (55.6)	.088
Hypertension (n, %)	46 (46.0)	57 (58.8)	.260	47 (59.5)	13 (72.2)	.071
Cardiovascular diseases (n, %)	7 (7.0)	18 (18.6)	**.015**			

Group 1, PEX without CVD; Group 2, PEX with CVD.

*P* values were calculated using Student’s *t*-test, Mann–Whitney *U* test, and χ^2^ as appropriate.

The bold indicate statistically significant *P* values.

CVD = cardiovascular disease; NLR = neutrophil-to-lymphocyte ratio; PEX = pseudoexfoliation syndrome.

**Table 2:** Laboratory findings of subjects with pseudoexfoliation syndrome and controls.

Lab findings	Control (n = 100)	PEX (n = 97)	*P* value	PEX without CVD; group 1 (n = 79)	PEX with CVD; group 2 (n = 18)	*P* value	*P* value	*P* value	*P* value
						Group 1 vs control	Group 2 vs control	Group 1 vs group 2	Control vs group 1 vs group 2
WBC count (10^3^/μL)	6.69 ± 1.56	6.70 ± 1.58	.943	6.69 ± 1.61	6.74 ± 1.49	.976	.891	.910	.991
Neutrophil count (10^3^/μL)	3.68 ± 1.15	3.92 ± 1.19	.145	3.87 ± 1.18	4.15 ± 1.23	.273	.118	.376	.230
Lymphocyte count (10^3^/μL)	2.26 ± 0.60	2.06 ± 0.53	**.018**	2.11 ± 0.55	1.87 ± 0.38	.092	**.009**	.083	**.017**
NLR	1.70 ± 0.56	1.97 ± 0.67	**.002**	1.90 ± 0.65	2.26 ± 0.68	**.048**	**.002**	**.023**	**.002**

Group 1, PEX without CVD; Group 2, PEX with CVD.

*P* values were calculated using Student’s *t*-test, Mann–Whitney *U* test, and χ^2^ as appropriate.

The bold indicate statistically significant *P* values.

CVD = cardiovascular disease; NLR = neutrophil-to-lymphocyte ratio; PEX = pseudoexfoliation syndrome.

PEX patients showed higher levels of NLR than the control group (*P* = .002; Table 2). When the PEX group was divided into groups 1 and 2 according to CVD, group 1 and group 2 showed significantly higher NLR levels than the control group 2 (*P* = .048 and *P* = .002, respectively; Table 2). Two-way ANOVA revealed the effect of PEX and CVD on the value of NLR, respectively. There was a statistically significant difference in NLR only by PEX (*P* = .019), not by CVD (*P* = .107). The interaction between PEX and CVD on NLR was not significant (*P* = .391).

## 4. Discussion

This study demonstrates that there is an association between PEX and elevated NLR regardless of cardiovascular comorbidity. NLR has been proposed as a simple and reliable biomarker for inflammatory conditions. Recent studies have also shown a prognostic role of NLR in patients with CVD.^[10,20–23]^ On the other hand, the incidence of CVD is known to be increased in PEX patients due to its underlying mechanism of PEX materials accumulating in vascular tissues resulting in impaired endothelial function and fibrillopathy.^[1,7–9,11,12]^ However, previous studies showing elevated NLR in PEX patients did not control the impact of CVD on the high NLR value.^[17,18]^ When comparing NLR by dividing the patient group to those with and without CVD, high NLR levels were confirmed not only in the group with CVD, but also in the group without CVD in the present study, confirming that NLR levels were elevated in all patients with PEX.

In patients with CVD, NLR is known as a factor that can predict cardiovascular event.^[20–23]^ There are 2 retrospective studies published on the association between PEX and elevated NLR.^[17,18]^ They reported significantly higher NLR in PEX and PXG patients than in controls. In the study by Kurtul et al,^[17]^ NLRs of control (n = 48), PEX (n = 55), and PXG (n = 19) groups were 1.51 ± 0.57, 2.08 ± 0.61, and 2.20 ± 0.58, respectively. In the study by Ozgonul et al,^[18]^ NLRs of control (n = 42), PEX (n = 34), and PXG (n = 29) groups were 1.84 ± 0.59, 2.33 ± 0.84, and 2.45 ± 0.82, respectively. These studies showed significant differences in NLR according to the disease severity of PEX. In this study, when the group of PEX patients was divided into PEX without glaucoma (1.99 ± 0.72; n = 56) and those with glaucoma (1.94 ± 0.60; n = 41) and compared with the control group (n = 100), the 2 PEX groups showed higher NLR values than the control group (*P* = .020 and *P* = .032), although there was no significant difference in NLR value between the 2 PEX groups (*P* = .950). However, in these previous studies, the number of subjects was small. Thus, there was a limit to the analysis results. Other risk factors such as cardiovascular morbidity that could raise NLR should be considered. In the present study, when PEX patients were divided into those with and without CVD, NLR showed a significant difference between the 2 PEX groups (1.90 ± 0.65 vs 2.26 ± 0.68; *P* = .023). The PEX without CVD group showed higher NLR than the control group (*P* = .048). Thus, PEX patients showed high NLR values regardless of cardiovascular morbidity, suggesting that attention should be paid to the interpretation of NLR levels when subjects have a CVD history. The two-way ANOVA results also indicate that PEX is the only factor that has a significant effect on NLR. That means whether the patient had CVD or not has no impact on the value of NLR. In general, it is known that the NLR is high in CVD, but the reason for the conflicting results in this study may be that the CVD ratio is relatively small compared with PEX due to the small number of controls.

The limitation of this study was that the number of subjects was relatively small, especially after PEX patients were divided into PEX with and without glaucoma. It is necessary to find out whether NLR level is different according to disease severity and prevalence of CVD. Ozgonul et al^[18]^ have also reported that the PXG group has higher NLR levels than the control and PEX groups. In addition, further study is needed to determine the mechanism of inflammatory factors that play roles in PEX pathogenesis. Since PEX is known to be associated with multifactorial biochemical processes involving growth factors and cytokines participating in subclinical inflammation,^[3–6]^ research through different stages will be needed, just as the development of therapeutic drugs is underway by approaching different levels of mechanisms in other diseases.^[24–26]^

In conclusion, this study suggested higher NLR values in PEX patients regardless of cardiovascular morbidity than in controls. Further studies on changes in inflammatory markers and related mechanisms are needed depending on risk factors such as cardiovascular morbidity and disease severity in PEX.

## Author contributions

Conceptualization: HYO and HYS.

Data collection: HYO, MYL, YCL, and HYS.

Formal analysis: HYO and HYS.

Methodology: HYO and HYS.

Supervision and validation: HYO and HYS.

Writing—original draft: HYO and HYS.

Writing—review and editing: HYS and HYO.

All authors have read and approved the manuscript.
